# Gastric and Duodenal Neuroendocrine Tumor Incidentally Found on Endoscopy During the Evaluation of Iron-Deficiency Anemia

**DOI:** 10.7759/cureus.22208

**Published:** 2022-02-14

**Authors:** Joyce Cheng, Ghanshyam Patel, Altaf Dawood, Ammar Aqeel

**Affiliations:** 1 Internal Medicine, Javon Bea Hospital—Riverside, Rockford, USA; 2 Internal Medicine- Gastroenterology, Javon Bea Hospital—Riverside, Rockford, USA

**Keywords:** iron deficiency anemia (ida), endoscopic ultrasound (eus), duodenal neuroendocrine tumor, gastrointestinal neuroendocrine tumor, gastric neuroendocrine tumor

## Abstract

Neuroendocrine tumors (NET) are a small fraction of overall gastrointestinal (GI) malignancies. Recently the incidence of NETs has increased due to advancements in diagnostic modality. While solid tumors are easily visible on routine endoscopy, identifying endocrine tumors can be difficult, and low incidence and non-specific presentation can be easily missed on upper gastrointestinal endoscopy (UGIE). The management differs based on the type of tumor and location, but the overall prognosis is good. We present a 59-year-old male with multiple NETs throughout the GI tract, diagnosed on repeat esophagogastroduodenoscopy (EGD) with endoscopic ultrasound (EUS) showing multiple gastric folds. A biopsy of multiple nodules was taken to diagnose type I NET with grade 2 differentiation finally. The mucosal nodules were resected with a band ligator, and surveillance endoscopy was recommended.

## Introduction

Neuroendocrine tumors (NET) arise from neuroendocrine cells that feature nerve cells and hormone-producing endocrine cells. These tumors can be functional or non-functional based on their hormone-like substance-producing capabilities, but all of them are considered cancerous. Nearly 175000 people live with NETs in the United States, with 12000 anticipated incidence each year [1]. NETs can begin in any part of the body, including the gastrointestinal (GI) tract (43%), lung (30%), and pancreas (7%) [1]. Stomach NETs are less than 4% of GI tract NETs and less than 2% of overall NETs [2]. This case report aims to be suspicious of stomach NETs when multiple gastric folds are noted on upper gastrointestinal endoscopy (UGIE) [3].

## Case presentation

A 59-year-old male with a significant past medical history of type II diabetes mellitus, essential hypertension, dyslipidemia, hypothyroidism, obstructive sleep apnea, deep vein thrombosis, and coronary artery disease status post stent placement to the left anterior descending artery (LAD) presented for outpatient endoscopic evaluation after being found to have iron-deficiency anemia. Earlier labs demonstrated decreased hemoglobin, decreased mean corpuscular volume (MCV), low Iron, and low ferritin with increased total iron-binding capacity, for which he was placed on an oral iron supplement. The patient's family history was significant for colon polyps with many siblings. Given the patient's family history and no hemoglobin improvement on the iron supplement, esophagogastroduodenoscopy (EGD) and colonoscopy were scheduled to evaluate the anemia further.

**Figure 1:**
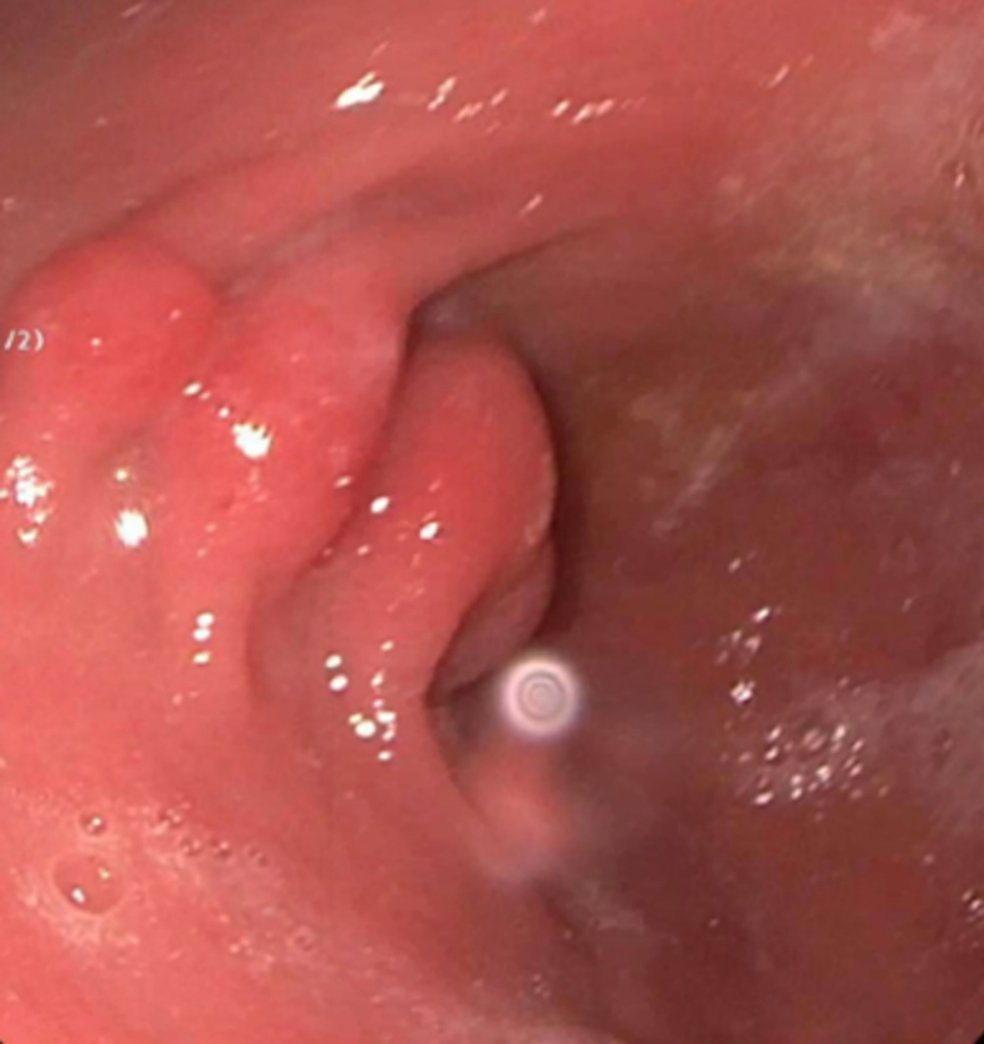
Initial Upper gastrointestinal endoscopy showing Gastric antrum with enlarged gastric folds and nodularity.

Initial esophagogastroduodenoscopy (EGD) findings showed a normal esophagus and duodenum; however, they noted an enlarged gastric fold (figure 1). Colonoscopy disclosed two polyps measuring 3-4 mm in the transverse and sigmoid colon. The biopsies of the gastric fold and polyps were obtained. Histopathology of the gastric biopsy showed mild chronic gastritis with reactive gastropathy; nonetheless, no evidence of acute inflammation, metaplasia, dysplasia, or malignancy was noted. The biopsy was negative for Helicobacter Pylori. The histopathology of transverse colon polyp revealed tubular adenoma without high-grade dysplasia or malignancy. The biopsy of a sigmoid colon showed a hyperplastic polyp.

**Figure 2:**
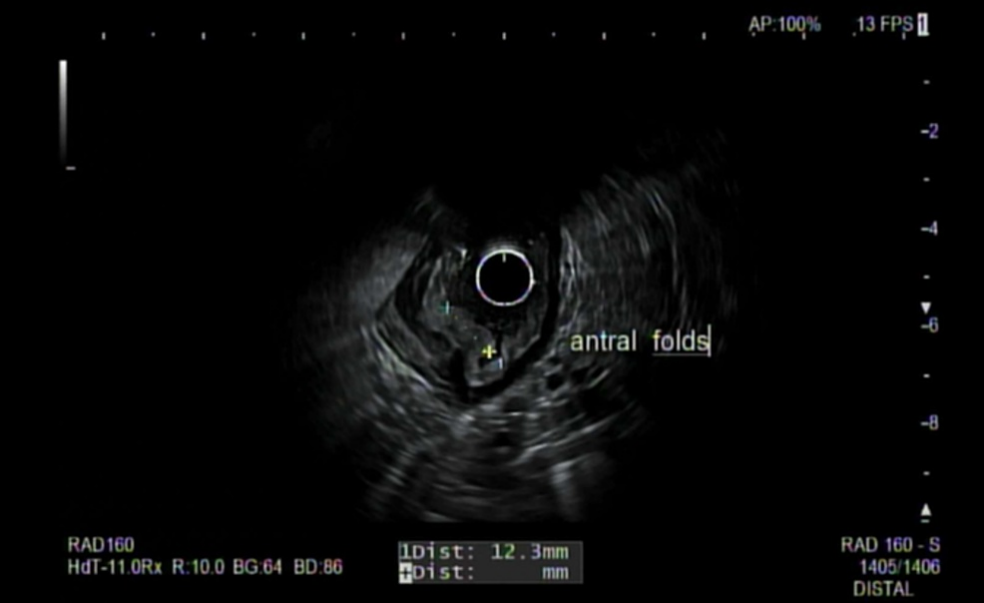
EUS exhibited localized wall thickening in the antrum of the stomach.

A repeat EGD was ordered with endoscopic ultrasound (EUS) to evaluate the enlarged gastric fold accurately. EUS findings showed a single 4 mm mucosal nodule in the gastric body and a 5 mm mucosal nodule in the duodenal bulb. Last enlarged gastric fold was again identified on EUS (figure 2) and precisely biopsied. Endosonographic findings of the enlarged gastric fold revealed thickening within the luminal interface/ superficial mucosa and deep mucosa.

The repeat histopathology of the enlarged gastric fold showed chronic atrophic gastritis and reactive gastropathy. The biopsies of the gastric body and duodenal bulb nodules showed well-differentiated type I NET (grade 2 of 3). The immunohistochemical analysis of tumor cells was positive for chromogranin A enterochromaffin-like cells (CgA ECL). Further labs showed increased gastrin, elevated parietal cell antibody, and decreased Vitamin B12 level. Hormone-producing tumor markers 5-Hydroxyindoleacetic Acid (5-HIAA) and morning cortisol were within the normal limit. CT chest/abdomen/pelvis showed no evidence of metastatic disease. Additionally, genetic testing was negative for multiple endocrine neoplasia type 1 (MEN1) syndrome.

After three months, a surveillance EGD with EUS illustrated an additional three 4 to 6 mm mucosal papules (nodules) within the gastric body and a single 6mm mucosal nodule in the duodenal bulb. With Roth net retrieval, all four mucosal nodules were resected via band ligator and snare mucosal resection. The patient was started on Vitamin B12 injections. The patient's hemoglobin was improved on follow-up, and surveillance of EGD with EUS was recommended every six months.

## Discussion

Neuroendocrine tumors (NETs) may arise in different organs, including the gastrointestinal tract, pancreas, lungs, gallbladder, thyroid gland, testes, ovaries, and skin. A small percentage of NETs are associated with inherited syndromes such as Multiple endocrine neoplasia type 1 (MEN1), Neurofibromatosis type 1 (NF-1), and Von Hippel-Lindau syndrome. Gastric NETs are differentiated into four types based on their clinicopathological characteristics. Gastric NETs type I to III comprise enterochromaffin-like cells (ECL), while type IV consists of endocrine cells.

70% to 80% of Gastric NETs are type I tumors that are associated with pernicious anemia, chronic atrophic gastritis (CAG), including autoimmune gastritis (AIG), and Helicobacter pylori-associated atrophic gastritis [1]. CAG causes loss of normal glandular cells leading to a decrease in acid production. This lack of acid causes hyperplasia of antral G-cells following hypergastrinemia. These enormous gastrin act as a trophic factor for ECL cells generating ECL hyperplasia. In our case, anti-parietal cell antibodies triggered hypergastrinemia, resulting in type I NETs. Vitamin B12 deficiency is seen among these populations due to the destruction of parietal cells from chronic gastritis.

Type I Gastric NETs have no specific symptoms; thus, they are found incidentally on EGD. However, the hormone-producing tumor may present with diarrhea, facial flushing, hyper or hypoglycemia, and GI ulcers. Type I Gastric NETs are multiple, less than 10 mm in diameter, and usually found on the gastric fundus or corpus [4,5]. On endoscopy, they portray as polypoid lesions or as smooth and rounded submucosal regions and may appear red or yellow [6]. Type I Gastric NETs are invaded to mucosa or submucosa of the stomach and have a very low risk of metastasis [4]. Type I tumor strongly positive for CgA ECL [7]. Though, in our case, it was borderline positive. These tumors are covered with normal mucosa; therefore, the biopsy with a deeper cut should be taken from multiple lesions, including antrum and fundus of the stomach [8].

National Comprehensive Cancer Network (NCCN) guideline version 2.2021 for management of NET patients suggests baseline EUS if clinically indicated, vitamin B12, and gastrin level check [9]. Following gastrin and CgA levels are not recommended. Type I Gastric NET management depends on their location, size, associated symptoms, and presence of metastasis. Tumors that are not metastasized or invaded muscularis propria are generally recommended for Endoscopic resection [8]. Management of advanced locoregional disease or metastases includes Octreotide, Everolimus, or Peptide Receptor Radionuclide Therapy with 177Lu-dotatete or cytotoxic chemotherapy. The surveillance endoscopy is recommended every 2-3 years or clinically indicated.

## Conclusions

Gastric NETs are difficult to diagnose; as it is asymptomatic and often missed on endoscopic evaluation. EGD with EUS can be a superior approach to diagnose NETs, and it is wiser to take multiple biopsy samples under EUS guidance during EGD to identify NETs. Here we present an interesting case of a middle-aged male who was found to have gastric and duodenal neuroendocrine tumor during repeat endoscopic evaluation for iron-deficiency anemia. This case emphasizes the importance of keeping NETs in differential while evaluating iron-deficiency anemia and the necessary evidence-based surveillance.
